# The Impact of Functional Movement Variability and Movement Creativity on Sport Climbing Performance

**DOI:** 10.1002/ejsc.70024

**Published:** 2025-07-19

**Authors:** Nikki Geerte van Bergen, Dominic Orth, Nicolas Deschle, Robert Berkenbosch, Marthe van der Toorn, Geert Savelsbergh, John van der Kamp

**Affiliations:** ^1^ Department of Human Movement Sciences Faculty of Behavioural and Movement Sciences Vrije Universiteit Amsterdam Amsterdam the Netherlands; ^2^ Amsterdam Movement Sciences Amsterdam the Netherlands; ^3^ Department of Health Sciences Brunel University of London London UK

**Keywords:** exploration, functional movement variability, movement creativity, movement repertoire, sport climbing

## Abstract

Expertise in sports is underpinned by the ability to adapt to changing individual, task and environmental constraints. The ecological dynamics approach positions movement variability as having functional properties thus enabling adaptation. Additionally, it holds that movement creativity emerges from movement variability in the process of exploration. To test these conjectures, we determined the relationships between movement variability, movement creativity and performance. Twenty‐one male climbers, ranging from experienced to high elite level participated. Functional movement variability and climbing performance were assessed in two different tests. The primary goal of the functional movement variability test was to perform a boulder problem in as many different ways as possible, whereas in the performance test, participants had six attempts to progress as far as possible. 2D hip position data (derived from video recordings using Kinovea) were collected to determine the number of distinct successful trajectories performed (movement variability), the degree of originality of each successful trajectory (movement creativity) and the trajectory length of the best attempt in the performance test (performance). Results revealed that both the ability to exhibit functional movement variability (*p* = 0.005) and the exploration of movement creativity (*p* = 0.002) were strongly associated with performance. Movement creativity contributed to performance in addition to movement variability (*p* = 0.024). We propose that variability is more than just the number of different movements; it should also be understood in how distinctly different these movements are, since they may reflect different patterns of exploration and determine the range of novel adaptations within an individual's capacity to be discovered.

## Highlights

- Movement variability as well as movement creativity are strongly associated with sport climbing performance.
- Movement creativity independently contributes to climbing performance beyond contributions of the functional movement variability.
- Movement variability is more than only the number of distinct movements (or the size of the movement repertoire) and also includes a distinction in terms of the breadth and depth of the differences.

## 1 Introduction

The main challenge of competitive climbing involves rapid and energy efficient adaptation to novel task constraints. Expertise is underpinned by the ability to adapt to the countless potential movement problems in different, sometimes creative, ways. Sport climbing, therefore, is an ideal platform to study associations between movement variability, creativity and performance.

Traditional views (Fitts and Posner 1967) regard movement variability as disruptive noise that should be minimised to prevent deviation from an optimal movement pattern. However, when the same task is repeated multiple times, movement patterns are never identical and also not at the elite level (Bartlett et al. 2007). Hence, the current ecological dynamics perspective positions movement variability as having functional properties enabling adaptation to changing constraints (Bartlett et al. 2007; Glazier and Davids 2009; Ranganathan and Newell 2013). During performance, movements are considered an emergent property of the interactions among constraints which set boundaries to and/or enable the movement solutions available (Newell 1986; Newell et al. 2003; Zahno and van der Kamp 2022). Athletes who can flexibly vary movement patterns to achieve the same movement solution can more effectively satisfy the interacting constraints on performance. Here, expertise is partly determined by an increased capacity for movement variability (van Bergen et al. 2023).

Adaptive behaviour in climbing has been identified as a key element of expert performance. Seifert et al. (2011) observed that expert ice climbers exhibit wider ranges of limb‐ and trunk movement compared to beginners. Additionally, the complexity of visual and movement exploration, which has been shown to increase with task difficulty (van Knobelsdorff et al. 2020), has been associated with better performance in novel tasks (Orth, Davids, and Seifert 2018). Similarly, being able to exploit a broad range of climbing movements was found to facilitate the learning process (Orth, Davids, Chow, et al. 2018). Furthermore, van Bergen et al. (2023) showed that the ability to exhibit high levels of grip strength across multiple hold types supports climbing performance. Finally, Künzell et al. (2021) observed that the ability to change the approach to a given movement problem in elite level boulder competitions resulted in significantly higher success rates. Collectively, these findings imply that a larger movement repertoire during performance is associated with more effective climbing. Possibly, a larger repertoire supports a more expansive exploration, increasing the opportunities through which individuals can fit to the demands of the environment. Another possibility is that these capacities enable novel movements to be discovered, conferring an additional performance benefit. Across the abovementioned studies, comparisons between skill levels have been made in terms of variability during performance, but they did not look into whether the available movement repertoire, as assessed separately from performance, predicts performance. This will be addressed in the current study.

Capacities for varying behaviour have also been studied extensively in adjacent fields (e.g., cognitive psychology) to understand creativity. Movement creativity has previously been described by Wyrick (1968) as the ability of individuals to produce original and functional movements. Cognitive approaches describe creativity as a personal ability or trait of an athlete (Memmert 2015; Richard et al. 2017). These studies define creativity as a person's ability to generate original ideas to solve given problems. In this line of reasoning, creativity is distinct from movement variability in the sense that distinctive neurocognitive processes underlie the discovery of novel original movements. In contrast, from an ecological dynamics perspective, movement creativity is considered as emerging from movement variability (Orth et al. 2017). Creative movements can be understood as new functional adaptations that satisfy the constraints of the encountered motor problem. Within the overall distribution of functional movement solutions, creative movements refer to solutions that are (statistically) rare and thus original (Simonton 2003). Exploration underpins the emergence of these functional and original motor solutions. Although movement creativity has been identified as a key element of expert performance in sports, its origins remains an issue of debate (Orth et al. 2017). There is some evidence that relates movement creativity to variability (Caso and van der Kamp 2020; Orangi et al. 2021; Orth et al. 2019), but again these studies assessed variability during performance within the same task and not independently.

Hence, in the present study we focus on the potential relationships between capacity for movement variability and performance. Separate variability and performance tests are performed. It is hypothesised that a large movement repertoire in the variability test enables climbers to apply a suitable solution to increasingly challenging movement problems encountered during the performance test. A large movement repertoire is typically considered in terms of the number of distinct solutions, an approach we follow in this study by considering the emerging movement trajectories. As it has remained unclear if movement variability and creativity have distinctive contributions to performance, exploratory analyses are performed to evaluate whether a larger movement repertoire is associated with increased movement creativity and whether this explains additional variance in performance. We expect that individuals who produce a high degree of movement variability also demonstrate more creative solutions on the variability test and score better on the performance test. However, since creativity is considered to emerge from movement variability, we do not expect movement creativity to explain any additional variance in performance.

## 2 Materials and Methods

### 2.1 Participants

Twenty‐one male sport climbers participated in the experiment (*M*_age_ = 27.62 ± 6.74 years, *M*_weight_ = 71.2 ± 5.9 kg, *M*_height_ = 180.3 ± 5.4 cm). The number of participants was based on a power analysis for correlation (*α* = 0.05, one‐tailed, effect size = 0.5, *β* = 0.8, required sample size = 21). Based on the smallest effect size of interest, as defined by Anvari and Lakens (2021), we intended to interpret observed results only in terms of strong effect sizes. Skill‐based inclusion criteria dictated a self‐reported climbing level in bouldering of advanced (IRCRA 18–23, 14 participants) to high elite (IRCRA 24 and higher, 7 participants), based on guidelines proposed by Draper et al. (2015). Participants were recruited based on their highest graded redpoint (i.e., route a climber can climb after the route has been previously rehearsed) within the last 12 months in bouldering. Climbing levels ranged from 20 to 30 with mean reported redpoint climbing ability levels corresponding to *M* = 23.0 ± 2.5. All participants signed informed consent before testing. The study was approved by the institution's ethics committee (Scientific and Ethical Review Board, Faculty of Behavioural and Movement Sciences, Vrije Universiteit Amsterdam (VCWE‐2018‐080)).

### 2.2 Equipment and Apparatus

**FIGURE 1:**
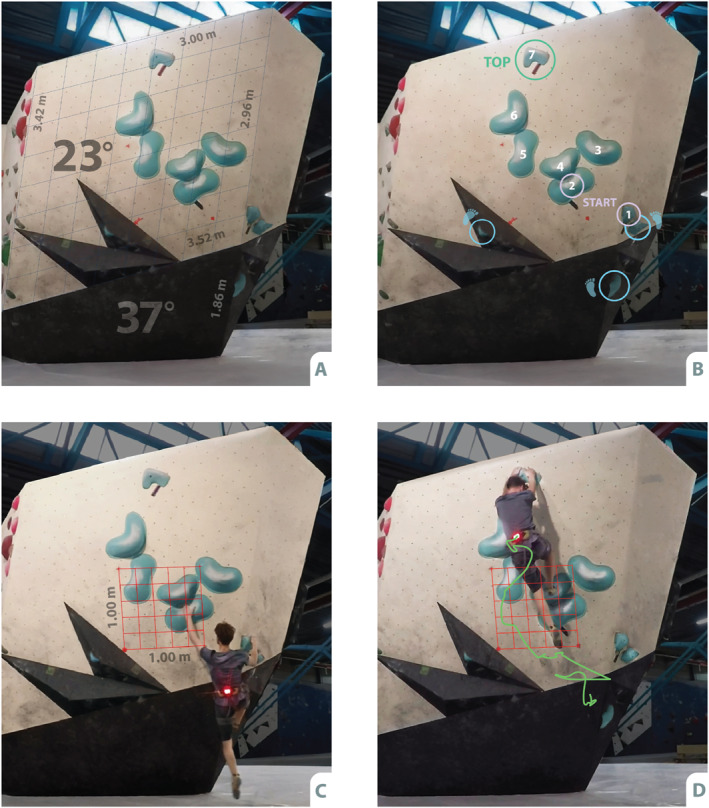
Visual representation of the variability test set‐up: (A) The wall angles and dimensions. The blue rectangular grid was placed over the main panel of the wall to show there is only very limited lens distortion after the Linear FOV correction (i.e., grid and upper and lower edges of the wall are largely aligned). (B) In light‐blue the starting holds, the top holds, seven hand holds, three footholds and two volumes; (C) The (red) perspective grid, connecting three markers in horizontal and vertical position in the centre of the wall, that was used to define a coordinate system in the plane of the wall for the *x*‐ and *y*‐position of the marker in terms of real‐world coordinates. The participant is situated in the starting position here, with the light bulb attached to the back being selected as a marker for semi‐automated tracking in Kinovea. (D) An example of a hip trajectory from a successful attempt on the variability test, tracked by Kinovea and expressed relative to the (red) perspective grid.

To assess climbing performance and movement variability, two climbing tests were performed in a set off section in a boulder gym (Boulderhal De Campus, Den Haag). Climbing attempts were filmed using a rearview camera (GoPro Hero4, resolution 1080p, framerate 60 fps) fixed to a standardised setup positioned at 0.80 m high and at a distance of 4 m to the wall. To avoid lens distortion, the camera was set at ‘Linear FOV’. The raster in Figure 1A shows that indeed only very minimal lens distortion remained with this method (i.e., the upper and lower parts of the wall are aligned with the rectangular grid). During the climbing tests, the position of the hip was filmed and tracked using a red‐light bulb attached at the participants' hip mid‐line (Figure 1C,D). Kinovea (version 0.8.27) was used to derive *x*‐ and *y*‐position data from the video recordings.

### 2.3 Experimental Setup

To examine the relationship between movement variability and climbing performance, two boulder routes were set up by an experienced route setter. To assess movement variability, a boulder problem with a difficulty level well below inclusion criteria was set up (Font 6A). The boulder consisted of seven light‐blue handholds, three footholds and two volumes (Figure 1B). These were set at a 23‐degree overhanging wall (on top of a 37‐degree basis, on which no handholds were placed, Figure 1A). The boulder problem was designed to invite many different trajectories or solutions. For the climbing performance test, a boulder problem with increasing difficulty was created, estimated to start as 7A and to finish as 8A (Font). The boulder problem contained 11 white handholds, forcing 12 hand movements and was situated on a 17‐degree overhanging wall without angle changes (not depicted).

### 2.4 Procedure

Separate tests were designed to independently assess functional movement variability and performance. These were performed in one session, lasting 2 h. When entering the climbing gym, participants received general instructions, signed informed consent and reported their age, weight, height and redpoint climbing level. Hereafter, participants proceeded to 15 min of self‐selected warm‐up exercises before receiving instructions for the functional movement variability test.

#### 2.4.1 Functional Movement Variability Test

Objectively capturing movement variability has proven to be challenging (Cortes et al. 2019). Studies investigating movement creativity have mostly assessed creativity using divergent thinking tasks, requiring athletes to think of as many solutions as possible without enacting them. Yet, ecological dynamics perspective holds that creative movement solutions typically manifest themselves in action while exploring the constraints, rather than as a priori cognitive idea. Hence, Moraru et al. (2016) modified a divergent thinking task into a divergent doing task to assess movement creativity. Accordingly, a divergent doing task was used in the current study as the functional movement variability test, where the primary goal was to solve a boulder problem in as many different ways (i.e., distinct trajectories) as possible within a 10‐min time span. Participants were encouraged to ‘try to think outside the box’. The difficulty level was set at least 4 IRCRA grades below the lowest reported climbing level to foster the exploration of different options. Based on pilot measurements, it was expected that 10 min would be enough to exhaust all possible solutions. Participants were informed that failed attempts did not count, but were not punished either.

Before starting the functional movement variability test, participants received 2 min observation time. Hereafter, participants were requested to put on a harness with a lightbulb attached to the back to track the hip trajectory and proceeded to their 10‐min test period. The use of magnesium was allowed.

#### 2.4.2 Climbing Performance Test

After finishing the functional movement variability test, participants rested for 15 min before proceeding to the climbing performance test. The test started with 2 min observation time, after which participants were allowed six attempts to finish the boulder problem with the objective to reach the top in as few attempts as possible. Between attempts, participants received 5 min of rest, during which they had to perform a cognitive task (solving a sudoku puzzle) to keep them from analysing their performance. In this way, all problem‐solving and exploration took place during the climb.

### 2.5 Data Processing

The dependent variables dictating the outcome of both the movement variability test and the climbing performance test were based on hip‐position data, derived from the video recordings made during both tests. Recordings were analysed using Kinovea (version 0.8.27), a video analysis programme that transformed the movements of the LED attached to the hip from the video fragments into 2D position data. To this end, the light‐bulb served as marker (Figure 1C) and was tracked semi‐automatedly by Kinovea (i.e., frame‐by‐frame confirmation was performed). In cases where Kinovea lost track of the marker, the last correct position was selected and the position of the marker was corrected manually frame‐by‐frame. The measurement frequency corresponds to the video frame rate (60 Hz).

Figure 1C,D show a red‐coloured perspective grid. This grid connects three markers that were attached to the centre of the wall, 1m apart in horizontal and vertical distance in order to set up a perspective grid in the plane of the overhanging wall in Kinovea and to translate the pixels in the image into real‐world coordinates. This process resulted in an output of Kinovea that consisted of x‐ and y‐coordinates of the hip over time and which was processed for further analyses using MATLAB.

#### 2.5.1 Functional Movement Variability

Adapting Moraru et al. (2016), functional movement variability was defined as the total number of distinct successful trajectories performed by each participant in the variability test (i.e., the number of different solutions to the boulder problem). A trajectory was defined successful when the final hold was controlled with two hands (IFSC Rules, see https://www.ifsc‐climbing.org/commissions/rules). In this definition, successful refers to one of the criteria for creative movement solutions, namely functionality (Orth et al. 2017), which implies that the emerging movement trajectory enables achieving the goal of the task. Unsuccessful trajectories were excluded from the dataset in order to meet the criteria of functionality. In total, 212 successful and 93 unsuccessful trajectories were performed. Among the unsuccessful trajectories three categories were distinguished: (1) a slip (22); (2) a solution that was successful in a later attempt (16); (3) others (55), which included trajectories that were seemingly impossible (or too difficult) for the participant to perform.

**FIGURE 2:**
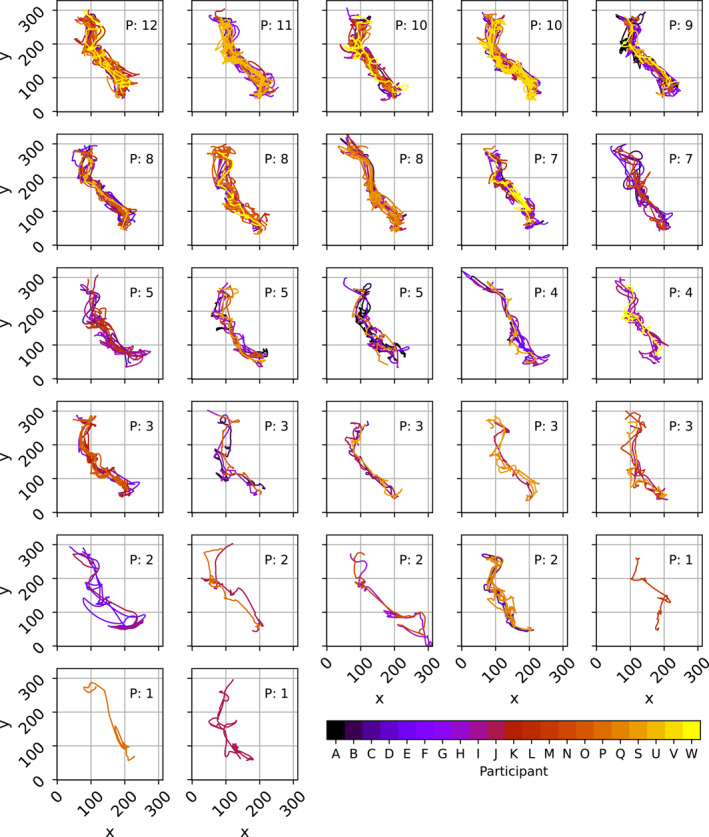
The different clusters identified. Colour‐coding shows which participant performed the trajectories depicted. Clusters are ordered based on the number of participants that performed them (i.e., clusters on the upper left are performed by most participants, clusters on the lower right are performed only by one participant). The number of participants that performed a cluster is mentioned in each cluster (‘P:’).

**FIGURE 3:**
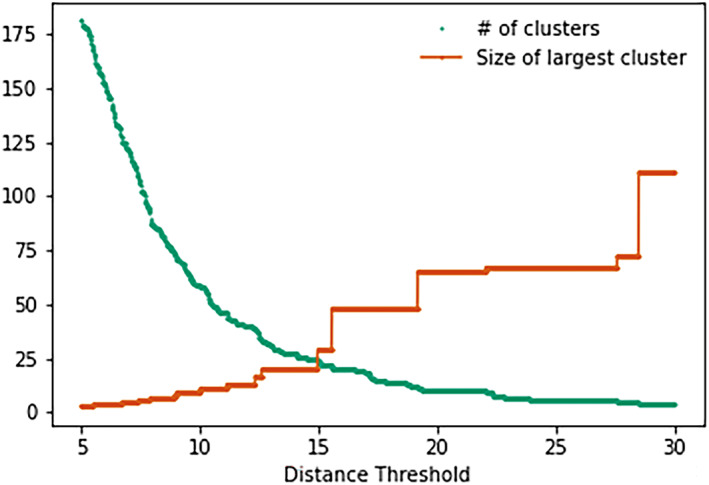
Visual representation of the number of clusters (green) and the size of the largest cluster (orange) as a function of different distance thresholds used for hierarchical clustering. The plateau in the number of clusters was used to determine the distance threshold. The size of the largest cluster steadily increases for thresholds smaller than 15, where it levels off and then increases stepwise. This point at 15, where the first large increase in the size of the largest cluster occurred was defined as the distance threshold.

Divergent thinking studies have often used intersubjective judgements to define originality (Hendry et al. 2018; Kempe and Memmert 2018; Memmert 2010; Zahno and Hossner 2023). Here, we followed a previously validated quantitative approach (Caso and van der Kamp 2020; de Joode et al. 2023; Moraru et al. 2016), using cluster analyses (Figure 2). All successful trajectories performed by all participants were included in this cluster analysis. First, the pairwise distance between all trajectories was computed using Symmetric Segment‐Path Distance (Besse et al. 2016). This measure is suitable because it is a shape‐based distance independent of the time index of the trajectory, and it considers the total length and the physical distance between trajectories (for a detailed description of the calculation procedure, see Besse et al. (2016)). Hereafter, hierarchical clustering was used to group the performed trajectories based on their proximity. This returned the whole hierarchy from one cluster per trajectory to only one cluster containing all trajectories. Next, the distance threshold at which to stop clustering was determined by exploring changes in the number of clusters (green line Figure 3) and the size of the largest cluster (orange line Figure 3) as a function of the distance threshold. For very large distance thresholds, the number of clusters becomes one, since all the trajectories are considered similar. By contrast, with very small thresholds, every trajectory is considered distinct. The balance was found by targeting the inflection point in the curve. The size of the largest cluster steadily increases for thresholds smaller than 15, where it levels off and then only increases stepwise. This point, where the first large increase in the size of the largest cluster occurred, was considered an appropriate data‐driven candidate for defining the distance threshold (set at fifteen).

Finally, for each participant, a functional movement variability score was determined by the number of distinct trajectories, that is, the number of different clusters the trajectories belonged to.

#### 2.5.2 Creativity

Creativity can be defined as a combination of functionality and originality (Orth et al. 2017). To meet the criterion of functionality, only successful trajectories were taken into consideration. To quantify creativity, the originality of these successful trajectories had to be determined. To this end, for each cluster (c) the prevalence was determined using Equation (1):

(1) $${\text{Prevalence}}_{c}=\frac{\#\;\text{participants}\;\text{showing}\;c}{\#\text{participants}}$$

Meaning that the most (M, Equation (2)) and least (L, Equation (3)) common clusters are given the prevalence scores,

(2) $${\text{Prevalence}}_{M}=\frac{\#\;\text{subjects}\;\text{that}\;\text{did}\;\text{cluster}\;M}{\#\text{subjects}}=\frac{21}{21}=1$$

(3) $${\text{Prevalence}}_{L}=\frac{\#\;\text{subjects}\;\text{that}\;\text{did}\;\text{cluster}\;L}{\#\text{subjects}}=\frac{1}{21}$$

The prevalence indicates how ‘original’ a cluster is. The originality of a cluster (c) was defined as 1/prevalence_c_. This means that every successful trajectory performed by a participant was now quantified based on the originality of the cluster it belonged to. Finally, for each participant the creativity score was calculated as the average of the originality scores of the successful trajectories performed by this participant.

#### 2.5.3 Climbing Performance

To define climbing performance, climbed distance in terms of trajectory length (in *x* and *y* direction) was calculated for each attempt. For this test, we were interested in how far participants progressed into the route. Therefore, the signal was smoothed using a second order lowpass Butterworth filter (cut‐off frequency 3 Hz) with the purpose of eliminating small deviations at the level of the hip, which may rather reflect exploratory behaviour rather than distance travelled. This ruled out that participants who explored and/or hesitated a lot at individual holds but did not progress far over the route would get similar trajectory length scores as participants who would progress far along the route without exploration and/or hesitation. The filtering frequency was determined by visual inspection. At 3 Hz small deviations were filtered out, but the highest point reached and the coarse path over the route were maintained. For each participant the trajectory length covered in the best attempt was used as a performance score. Two participants topped and thus reached the highest possible performance score.

### 2.6 Statistical Analysis

The Kolmogorov–Smirnov test was used to test for normality, showing that only the creativity score was normally distributed, whereas the other variables were not. Therefore, Spearman's rho (*ρ*) was used to test for correlations between the number of different clusters, creativity, climbing level and performance. Correlation coefficients were interpreted only in case of a strong relationship, that is, for values of ±0.5 and higher (Field (2009)). Stepwise hierarchical regression was performed to explore the contribution of creativity on top of the number of different clusters in predicting climbing performance. The number of different clusters was entered in the first step, and creativity in the second step. Significance level was set at *p* < 0.05. In the second step, coefficients were only interpreted if the change in *R* ^2^ for this step was significant.

## 3 Results

**FIGURE 4:**
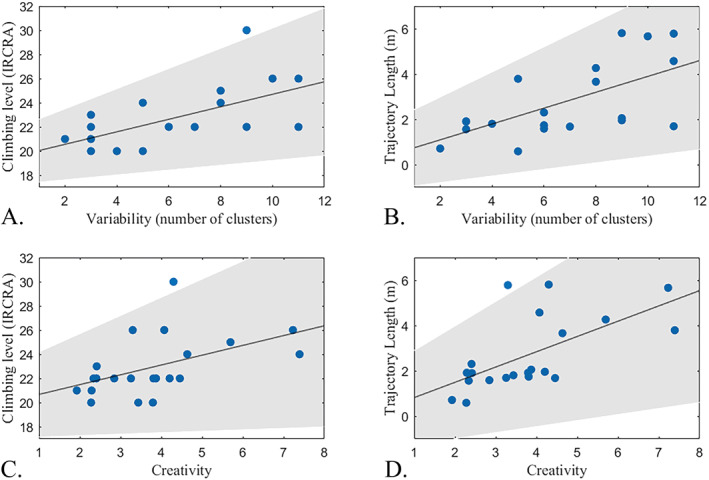
The relationship between (A) The number of different clusters and climbing level. (B) The number of different clusters and trajectory length. (C) Creativity and climbing level. (D) Creativity and trajectory length. Shaded areas give the 95% confidence intervals. Note that, as a result of overlapping datapoints, not all participants are visually distinguishable in panels (A and B).

The number of different clusters was significantly and strongly correlated to both climbing level (*ρ* = 0.650, *p* = 0.001) and climbing performance (*ρ* = 0.592, *p* = 0.005) (Figure 4A,B).

A strong significant positive correlation was found between creativity and climbing level (*ρ* = 0.612, *p* = 0.003). Creativity was also strongly and significantly correlated to trajectory length (*ρ* = 0.640, *p* = 0.002) (Figure 4C,D).

**FIGURE 5:**
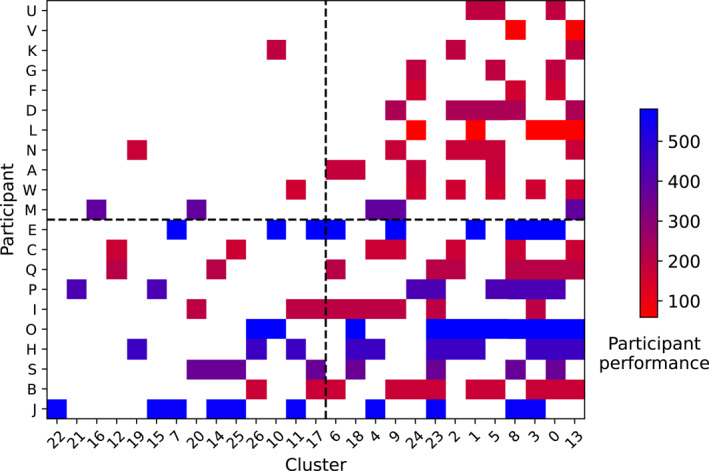
Representation of the clusters performed by each participant. Participant are ranked based on the number of clusters performed (lowest ranked participant (J) performed most clusters, highest ranked participant (U) performed least). The clusters are ranked on their prevalence and thus creative value (i.e., clusters on the left are least common thus most creative, clusters on the right are least creative). Finally climbing performance of each participant is added by colour‐coding. Blue represents high‐performance and red represents low‐performance.

The number of different clusters and creativity were significantly and strongly correlated (*ρ* = 0.529, *p* = 0.014). Next, for each participant, the number of different clusters performed and the associated creativity scores were visualised. Figure 5 shows the clusters (i.e., the groups of distinct trajectories) on the *x*‐axis and the participants on the *y*‐axis. The participants are ordered based on the number of different clusters from which they performed trajectories (i.e., participants at the bottom of the plot performed trajectories from the highest number of clusters). The clusters are ordered based on their creativity score (i.e., clusters in left half of the graph are more creative and performed by few participants, whereas clusters on the right half are less creative and shown by almost all participants). The clusters with the highest creativity scores were indeed performed by the participants who performed the highest number of clusters (Figure 5, bottom left quarter). This included the best performing participants (in blue). The participants who showed trajectories from a few clusters only tended to perform clusters that are less creative (Figure 5, upper right). These participants also tended to show lower performance scores (red).

Finally, the degree to which creativity adds to the prediction of performance in combination with the number of different clusters was explored. The colour‐coding in Figure 5 indicates that participants performing trajectories from many clusters (i.e., multiple distinct trajectories) indeed were the best performers (all blues are represented in the lower half of Figure 5). However, looking at the left side of Figure 5, the most creative clusters tended to be more often but not exclusively performed by the best performers (i.e., blues are more prevalent in the left half of Figure 5). Stepwise hierarchical regression was performed to predict climbing performance based on the number of different clusters in the first step (Step 1: *β* = 0.635, *r* ^2^ = 0.403, *F* = 12.837, *p* = 0.002). Then in the second step, creativity was added to the regression model, resulting in a significantly improved model fit (Step 2: *β*_number of clusters_ = 0.453, *β*_creativity_ = 0.429, Δ*r* ^2^ = 0.151, Δ*F* = 6.107, *p* = 0.024).

## 4 Discussion

The objective of this study was to evaluate associations between movement variability, creativity and climbing performance. Since expertise in competitive climbing is underpinned by the ability to adapt to novel task constraints in various, and often creative ways, both a high degree of movement variability and high creativity were expected to be related to better climbing performance. However, because we considered movement creativity as emerging from movement variability, we anticipated that creativity would not explain additional variance in performance. We found that both variability and creativity were associated strongly with performance, confirming the first hypothesis. Interestingly, the regression analysis revealed that creativity did significantly add to the explained variance in performance beyond variability—perhaps suggesting two complementary avenues through which individuals can prepare for performance under novel constraints.

### 4.1 A Larger Repertoire Supports Novel Task Performance on the Basis That It Increases the Opportunities of Performer‐Environment Fit

Skilled performance requires an athlete to functionally adapt their motor actions to shifting constraints, exhibiting degenerate behaviour. Degeneracy implies that an athlete can vary motor actions without compromising function (Edelman and Gally 2001). Increased degeneracy leads to an increased cooperation between intrinsic dynamics and task constraints. It appears that when a large range of movement patterns is available, individuals can utilise their inherent degeneracy to more consistently achieve the task goal (Komar et al. 2015; Rein et al. 2010). Being able to adapt to task goals under various circumstances is considered a prerequisite for sport performance (He et al. 2023; Robalo et al. 2021). This is supported in our results, where the ability to exhibit variable motor behaviour was linked to performance.

Movement variability is often considered an important factor underpinning performance. Most studies, however, have focussed on equating skill level with performance outcomes, rather than determining the underpinning behaviours characterising performance. The exact role movement variability plays in performance remains an issue of debate. Multiple studies show that expert performers can exhibit different coordination patterns to achieve the same task (Andrews et al. 2024; Komar et al. 2015; Rein et al. 2010). It is assumed that these differences in the selected motor actions originate from the capability to explore in the face of changing constraints to quickly find a solution (Orth et al. 2017). In this line of reasoning, the current movement repertoire could be considered as an impression of outcomes of exploration over time. Demonstrating a large movement repertoire to match internal and external constraints was identified by experts as a key performance parameter in climbing (Sanchez et al. 2019). However, this conclusion was drawn based on interviews and not bolstered with experimental results. In fact, we could not find any study that separately assessed movement variability and subsequently linked that to performance in another task or situation. Our study contributes to the existing literature on movement variability by directly predicting performance in sports from functional movement variability.

### 4.2 Creative Movements Are Associated With Enhanced Performance Because They Emerge From Exploration‐Induced Increases of the Regular Movement Repertoire

In ecological dynamics, creative movements are functional and statistically rare and emerge when movement variability is high (Hristovski et al. 2012; Orth et al. 2017; Simonton 2003). We find, as predicted from this theorising, there is indeed a strong correlation between the extent of movement variability and creativity. These two variables are conceptually linked through exploration. Exploration is an individual's search for adaptive solutions that satisfy the constraints on action. Hence, continuous exploration results in enhanced movement variability, among which also novel creative movement solutions may emerge that go beyond the regular movement variability or repertoire (Hristovski et al. 2011, 2012). For instance, in an intervention study, Zahno and Hossner (2020) showed that training focussed on increasing movement variability of youth football players led to more creative actions on the field. With an increase in exploration‐induced movement variability, the likelihood of creative movements emerging also increases. Since a larger movement variability underpins performance, it is perhaps unsurprising to find that movement creativity is also strongly associated with better performance, as predicted by Orth et al. (2017). The findings of the current study add to the limited base of empirical evidence linking creativity to performance in sports.

### 4.3 Implications of Movement Creativity Having Independent Contributions to Performance

**FIGURE 6:**
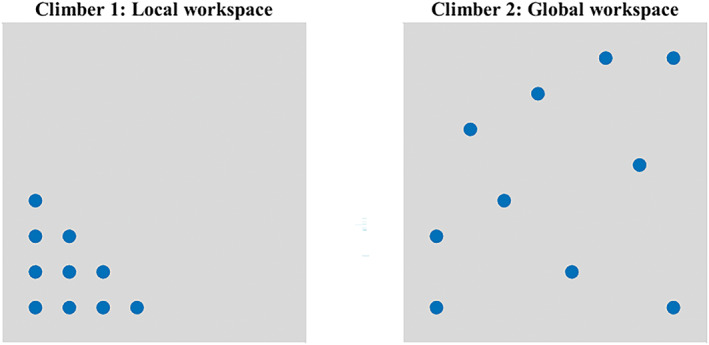
Visual representation of two hypothetical workspaces. Both climbers performed the same number of different trajectories (10), but for the first climber these trajectories are all situated in the same corner of the workspace (local), whereas for the second climber the trajectories are widely spread (global). These differences influence the range of the performance landscape that is explored.

Summarising, creative movements have been identified as emerging from movement variability, as they more likely manifest themselves within a larger movement repertoire (Davids et al. 2015; Hristovski et al. 2011; Orth et al. 2017). In fact, since movement creativity emerges from movement variability, it was not anticipated to have additional contributions to performance. Nonetheless, we found that creativity did contribute to performance beyond movement variability suggesting an alternative mechanism, related to novelty, through which it may do so. Importantly, we quantified movement variability in terms of the amount of different motor solutions (i.e., distinct trajectories). However, movement variability can be conceived in more ways than only the number of different solutions. For example, the proximity or difference between solutions can also differ in magnitude. To illustrate, imagine two hypothetical cases (outlined in Figure 6). A first participant performs 10 clusters that are all different, but the proximity is relatively small. Within a performance landscape, this scenario implies that the clusters are all situated locally, meaning that the next solution is always explored in the small neighbourhood of previous solutions (Gel'fand and Tsetlin 1962). A second hypothetical participant also performs 10 different clusters, but these are more globally distributed across the landscape (according to Gel'fand and Tsetlin (1962), and these non‐local methods are defined by a non‐continuous search trajectory. Instead of exploring only a small neighbourhood, the method allows jumps to distant regions in the parameter space). Although both participants would score similarly on the current movement variability test (performing trajectories within the same number of different clusters), their movement repertoire would clearly differ. The local search strategy (Newell and McDonald 1992) of the first participant may result in a deep, relatively narrow repertoire, whereas the second participant's global search strategy may be associated with a broad movement repertoire. Subsequent research should therefore also consider the degree of proximity of the distinct movement solutions when quantifying movement variability. In the same vein, movement creativity may differ in terms of being rare novel local or global adaptations, not unlike the distinction between persistent and flexible creativity by Nijstad et al. (2010). All this would also require to re‐assess whether movement creativity is an independent predictor of performance or not.

Having said this, the current finding that movement variability and motor creativity contribute independently to performance has implications for training practices. Many training programmes aiming to develop creativity in sports have been focusing on improving divergent thinking in athletes (Memmert 2015). Zahno and Hossner (2020) provided evidence that increasing movement variability was more effective for exhibiting creative actions than a training programme focussed on enhancing divergent thinking. Our results indicate that functional movement variability and creativity are strongly correlated, advocating for methods that primarily target movement variability. However, the current findings also suggest that higher levels of movement creativity additionally support performance, indicating that also promoting creativity may facilitate performance. Both larger movement variability as well as larger movement creativity could be induced by designing training conditions that encourage exploration. Whether this should be global or local may depend on individual cognitive (e.g., working memory, Orth et al. (2017)) and physical constraints (e.g., grip strength, van Bergen et al. (2023)). For now, a training programme aimed at diversifying the search strategies that an athlete can exploit might be a fruitful avenue to pursue.

### 4.4 Limitations and Future Research

Taken together, our findings strengthen an ecological dynamics approach on movement variability and creativity. Considering the small base of empirical evidence for this conceptualisation of variability in sports, the results obtained in this study need to be replicated in other situations to claim wider generalisability. We have presumed that variability emerges from exploration, but we have only looked at impressions of the outcomes, not how they unfold over time. If indeed the way performers explore is critical, then it would be crucial for future research to take this temporal dimension into account as well.

Furthermore, as a first study looking into creativity in climbing, we decided to measure movement variability, creativity and performance in terms of climbing trajectories. Following previous work (Hacques et al. 2022; Legreneur et al. 2019; Orth et al. 2014), we analysed these in a 2D plane. Considering the research setup and the design of the wall used for the variability test, conducting the analysis in 3D instead of 2D would have been a more accurate alternative. As such, the 2D analysis can be considered a limitation of the current study. Not only would 3D analysis be more accurate, especially in the context of different wall inclinations, but it would also enable considering more aspects of movement variability such as hip‐to‐wall distance and hip roll. More importantly perhaps, we would not only address movement trajectories but also examine creativity at the level of the actions with the hands and feet. Previous studies showed that climbers can move the hip without moving hands or feet, and conversely, hand or feet movements do not always result in displacements at the level of the hip (Boulanger et al. 2016; Seifert et al. 2018). Therefore, movement variability and creativity in climbing need to be addressed at these multiple levels.

## 5 Conclusion

The present results demonstrate that climbing performance is associated with the ability to both exhibit variable and creative movements, as is predicted from an ecological dynamics approach (Hristovski et al. 2011; Orth et al. 2017). However, although the ecological dynamics approach argued that performance is mainly grounded in functional movement variability, we found that creativity independently contributed to performance. We therefore proposed an amended view on how functional movement variability underpins performance, in which movement variability not only encompasses the number of distinct movements (or the size of the movement repertoire) but also includes the proximity or breadth and depth of the differences. These differences are induced by different types of exploration, which may also determine the range of novel and creative adaptations within reach to be discovered.

## Ethics Statement

We declare that this study is submitted solely to European Journal of Sport Science and that it is not under review nor published elsewhere.

## Consent

All participants signed informed consent before testing and the study was approved by the institution's ethics committee (Scientific and Ethical Review Board, Faculty of Behavioural and Movement Sciences, Vrije Universiteit Amsterdam, VCWE‐2018‐080).

## Conflicts of Interest

The authors declare no conflicts of interest.

## Data Availability

The data that support the findings of this study are available from the corresponding author upon reasonable request.
